# Long Noncoding RNA uc001pwg.1 Is Downregulated in Neointima in Arteriovenous Fistulas and Mediates the Function of Endothelial Cells Derived from Pluripotent Stem Cells

**DOI:** 10.1155/2017/4252974

**Published:** 2017-12-13

**Authors:** Lei Lv, Haozhe Qi, Xiangjiang Guo, Qihong Ni, Zezhen Yan, Lan Zhang

**Affiliations:** Department of Vascular Surgery, Ren Ji Hospital, School of Medicine, Shanghai Jiao Tong University, Shanghai, China

## Abstract

Recent studies indicate important roles for long noncoding RNAs (lncRNAs) as essential regulators of gene expression. However, the specific roles of lncRNAs in stenotic lesions of arteriovenous fistula (AVF) failure are still largely unknown. We first analyzed the expression profiles of lncRNAs in human stenosed and nonstenotic uremic veins using RNA-sequencing methodology. A total of 19 lncRNAs were found to be differentially expressed in stenotic lesions. Among these, uc001pwg.1 was one of the most significantly downregulated lncRNAs and enriched in both control vein segments and human umbilical vein endothelial cells (HUVECs). Further studies revealed that uc001pwg.1 overexpression could increase nitric oxide synthase (eNOS) phosphorylation and nitric oxide (NO) production in endothelial cells (ECs) derived from human-induced pluripotent stem cells (HiPSCs). Mechanistically, uc001pwg.1 improves endothelial function via mediating MCAM expression. This study represents the first effort of identifying a novel candidate lncRNA for modulating the function of iPSC-ECs, which may facilitate the improvement of stem cell-based therapies for AVF failure.

## 1. Introduction

Patients with end-stage renal disease rely on hemodialysis, which requires a vascular access providing high blood flow rates preferably achieved through an AVF conduit [1]. However, the problems associated with vascular access dysfunction are the most common reason for increased morbidity, mortality, and length of in-hospital stay for patients and represent major clinical, social, and financial burden even for the developed countries [2, 3]. Venous neointimal hyperplasia (NH) is the predominant cause of stenosis.

Histological investigations have confirmed that venous neointimal hyperplasia is the predominant cause of stenotic lesions of AVF failure [4]. AVF stenosis occurs at the outflow vein due to venous neointimal hyperplasia and results in the failure of 60% of AVFs within 2 years [5]. Currently, there are no prophylactic treatments to ameliorate the progression of neointimal hyperplasia in AVFs. Percutaneous transluminal angioplasty for stenosis in functioning forearm AVF has been found to significantly improve patency and decrease access-related morbidity [6]. However, the disadvantages of these procedures are that they require frequent revision as the 12-month patency rate can be as low as 26% [7]. At present, there is minimal understanding of pathological and molecular mechanisms in AVF failure. The endothelial cell (EC) monolayer is at the interface between the extravascular space and blood, playing a crucial role in the modulation of vascular homeostasis [8]. Endothelial dysfunction has been implicated as an early step in the pathogenesis of neointima [9]. Therefore, improving endothelial function is critical for the prevention and treatment of neointima.

Long noncoding RNAs (lncRNAs) represent a diverse type of long RNA molecules lacking protein-coding capacity, with a length of larger than 200 nucleotides [10, 11]. A growing body of work has proved that lncRNAs play essential roles in a variety of biological processes, such as cell growth, differentiation, and immune response. However, insufficient information is available about the effect of lncRNAs in the context of AVF failure and about the role of lncRNAs in endothelial function.

In the current study, we examined the expression profiles of lncRNAs in stenosed vein segments of primary AVFs from uremic patients via RNA-sequencing analysis. In addition, we identified an unannotated lncRNA, uc001pwg.1, which positively regulates the function of a stem cell type, ECs derived from human-induced pluripotent stem cells (HiPSCs), which is more applicable for future translational and clinical research. During this process, uc001pwg.1 regulates the expression of melanoma cell adhesion molecule (MCAM). Based on these findings, we suggest that uc001pwg.1 may offer an attractive target for improving AVF function in uremic patients.

## 2. Material and Methods

### 2.1. Patients and Tissue Samples

This study was given ethical approval by the Renji Hospital Ethical Committee, Shanghai, China. All participants provided written informed consent to participate in this study. Stenosed vein segments were harvested from the primary AVFs just distal to the anastomosis at the time of surgical revision in 4 patients. Control vein segments were harvested from 3 predialytic patients at the time of their first operation for vascular access [12]. The two groups were statistically similar in sex and age. A detailed description of the two groups is provided in Supplementary Table 1.

### 2.2. RNA-Sequencing Analysis and Gene Ontology Analysis

LncRNA-Seq high-throughput sequencing and subsequent bioinformatics analysis were all done by CloudSeq Biotech (Shanghai, China). Briefly, paired-end reads were harvested from Illumina HiSeq 4000 sequencer and were quality controlled by Q30. After 3′ adaptor-trimming and low quality reads removing by the cutadapt software (v1.9.3), the high-quality trimmed reads were aligned to the reference genome (UCSC HG19) guided by the Ensembl GFF gene annotation file with the hisat2 software (v2.0.4). Then, the cuffdiff software (v2.2.1, part of cufflinks) was used to get the gene level FPKM as the expression profiles of lncRNA, and fold change and *q* value were calculated based on FPKM, and differentially expressed LncRNAs were identified. Differentially expressed lncRNAs with statistical significance were identified through volcano plot filtering and fold-change filtering. Finally, hierarchical clustering was performed based on differentially expressed lncRNAs using Cluster Tree view software (Stanford University, Palo Alto, CA, USA). A gene ontology (GO) analysis was performed to characterize genes and gene products in terms of the biological process, cellular component, and molecular function. Fisher's exact test was used to find if there was overlap between the differentially expressed list and the GO annotation list.

### 2.3. Generation of iPSC-ECs

HiPSCs were generated in our laboratory previously. HiPSCs are routinely maintained on Matrigel (BD, 356234)-coated plates in TeSR-E8media (Stem Cell) and passaged mechanically. Differentiation will be induced two days after passaging colonies by replacing TeSR-E8 medium with differentiation media based on *α*-MEM (Gibco) and timed addition of the following factors: 25 ng/ml Activin A (PeproTech, AF-120-14E), 30 ng/ml bone morphogenetic protein (BMP) 4 (PeproTech, AF-120-05ET), 50 ng/ml VEGF 165 (R&D Systems, 293-VE), and the small molecule inhibitor CHIR99021 (Selleck, S1263). On day 3 and day 7 of differentiation, the medium will be refreshed with *α*-MEM containing 50 ng/ml VEGF and 10 *μ*mol/L SB43152 (PeproTech, 1614) only. Single-cell suspensions were incubated with PE-conjugated anti-human CD31 antibody, and flow cytometry was used to purify the ECs. The purity of the HiPSC-EC was >90% by phenotype and CD31 immunostaining.

### 2.4. Cell Culture

Human umbilical vein endothelial cells (HUVECs), umbilical vein smooth muscle cells (HUVSMCs), and human pulmonary artery fibroblasts (HPAFs) were purchased from ScienCell (Carlsbad, CA, USA) and were cultured in fully supplemented endothelial growth medium (EGM-2, Lonza, Walkersville, MD, USA), smooth muscle cell medium (SMCM, ScienCell), and fibroblast medium (SMCM, ScienCell), respectively.

### 2.5. HiPSC-EC Transduction

Adenoviral vectors containing uc001pwg.1 and control adenoviruses were purchased from GeneChem Inc. (GeneChem, Shanghai). HiPSC-ECs were grown in EC growth medium (cat. number MCDB-131C, Vec Technologies) to 80% confluence and treated with adenoviruses containing uc001pwg.1 or control adenoviruses. Eighteen hours after adenoviral transduction, fresh media was added to the cells, and 3 days after transduction, transfection efficiency was confirmed by qRT-PCR.

### 2.6. Quantitative Real-Time PCR (qRT-PCR)

Total RNA was extracted from samples using TRIzol reagent (Invitrogen) and converted into cDNA using the Fermentas RT kit according to the manufacturer's instructions. PCR was performed in a total reaction volume of 25 *μ*L, containing 12.5 *μ*L SYBR Premix Ex Taq (2x), 2 *μ*L cDNA, 1 *μ*L forward primer (10 *μ*M), 1 *μ*L reverse primer (10 *μ*M), 0.5 *μ*L ROX Reference Dye II (50x), and 8 *μ*L double-distilled water. Amplification efficiency was evaluated via standard curve analysis. All samples were normalized to GAPDH, and the experiment was repeated three times. The following primers were used:

uc001pwg.1, forward: 5′-GCTGTGATTGTGTGCATCCT-3′, reverse: 5′-GAAGAGTGAGCAGGGAGCTG-3′; GAPDH, forward: 5′-GGCCTCCAAGGAGTAAGACC-3′, reverse: 5′-AGGGGAGATTCAGTGTGGTG-3′.

### 2.7. Western Blotting

The primary antibodies against MCAM (1 : 1000), eNOS (1 : 1000), phosphorylation eNOS (Ser^1177^) (1 : 1000), and GAPDH (1 : 1000) were purchased from Cell Signaling Technology (Danvers, MA). Western blot analyses were carried out as previously reported [12].

### 2.8. Detection of NO

NO release was measured by using DAF-FM diacetate. Briefly, HiPSC-ECs were seeded on glass coverslips. 48 h after transduction, the cells were incubated with DMEM containing DAF-FM (5 *μ*M) for 30 min in the dark at 37°C and then washed with PBS. Images were obtained using fluorescence microscopy (Olympus America Inc., NY, USA).

### 2.9. Statistical Analysis

Results are expressed as mean ± standard deviation. The data were monitored using two-tailed *t*-test and chi-square test as appropriate. Data analyses were performed using GraphPad 5.0 software. The significance threshold was defined by a *P* value of <0.05.

## 3. Results

### 3.1. uc001pwg.1 Is Downregulated in Stenotic Veins of AVF

**Figure 1:**
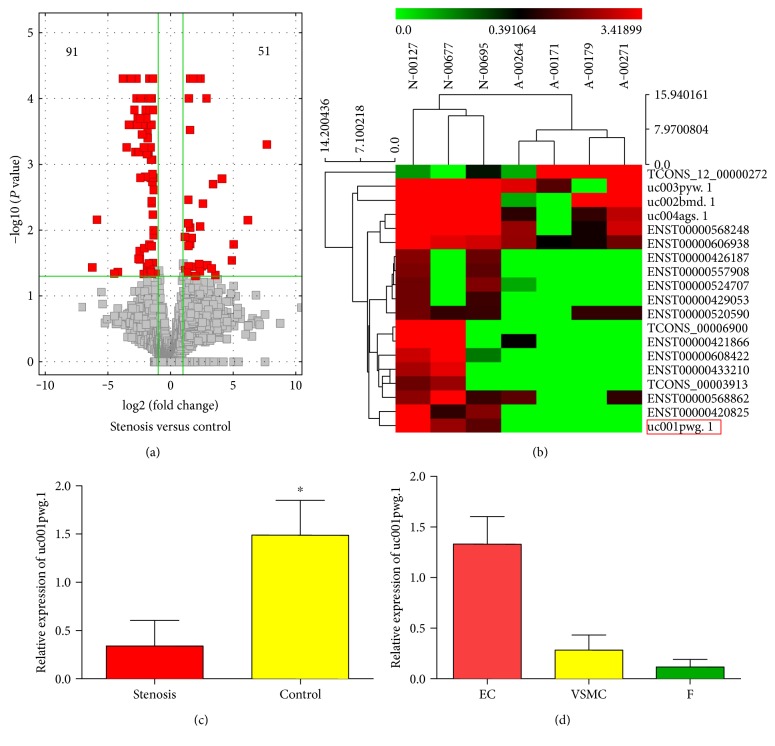
Long noncoding RNA (lncRNA) profiles differentiate the stenosed vein segments of arteriovenous fistulas (AVFs) from the control segments. (a) A volcano plot provided the fold change and *P* values of differentially expressed lncRNAs. The vertical lines represent a 1.5-fold change in expression (up or down), and the horizontal lines represent *P* values = 0.05. (b) Heatmap of selected aberrantly expressed lncRNAs in the stenosed vein segments of AVF and the controls. Colors indicate relative signal intensities: red and green colors indicate upregulated and downregulated lncRNAs, respectively. (c) Verification of uc001pwg.1 by quantitative reverse-transcription polymerase chain reaction (qRT-PCR) in the stenosed vein segments of AVF and the controls. (d) qRT-PCR analysis of uc001pwg.1 in different human vessel cells (VSMC: vein smooth muscle cells; EC: endothelial cells; F: fibroblasts). Triplicate assays were done for each RNA sample, and the relative amount of uc001pwg.1 was normalized to GAPDH. Values are expressed as mean ± standard deviation. ^∗^*P* < 0.05.

To explore the potential biological functions of lncRNAs in stenotic lesions of AVF failure, we examined the expression patterns of lncRNAs in stenotic veins of AVF and control veins. As shown in Figure 1(a), 142 lncRNAs were observed as differentially expressed, with 51 lncRNAs upregulated and 91 lncRNAs downregulated in the stenosis group compared to the control group. Detailed information regarding the differentially expressed lncRNAs is shown in Supplementary Table 2. The aberrantly expressed lncRNAs were further subjected to GO analysis (Supplementary Figure 1). Our data showed that some genes related to cell adhesion and junction, cell migration, membrane raft, and so forth were significantly enriched.

Afterward, we removed lncRNAs that were not expressed in the mainly same trend. We kept lncRNAs with at least a threefold change, *P* < 0.05 and FPKM > 0.1 in at least 2 samples. Finally, 19 lncRNAs which met our strict inclusion criteria were selected. Organization of the expression profiles into heatmaps better describes the expression patterns of lncRNAs (Figure 1(b)). Among the decreased lncRNAs, we identified a novel lncRNA (named uc001pwg.1), which was located on chromosome11 (119179240-119192231), enriched in both control vein segments and HUVECs (Figures 1(c) and 1(d)). Consistent with the microarray data, uc001pwg.1 is shown to be significantly suppressed in stenotic veins of AVF using qPCR (Figure 1(c)).

### 3.2. uc001pwg.1 Enhances the Function of HiPSC-ECs

**Figure 2:**
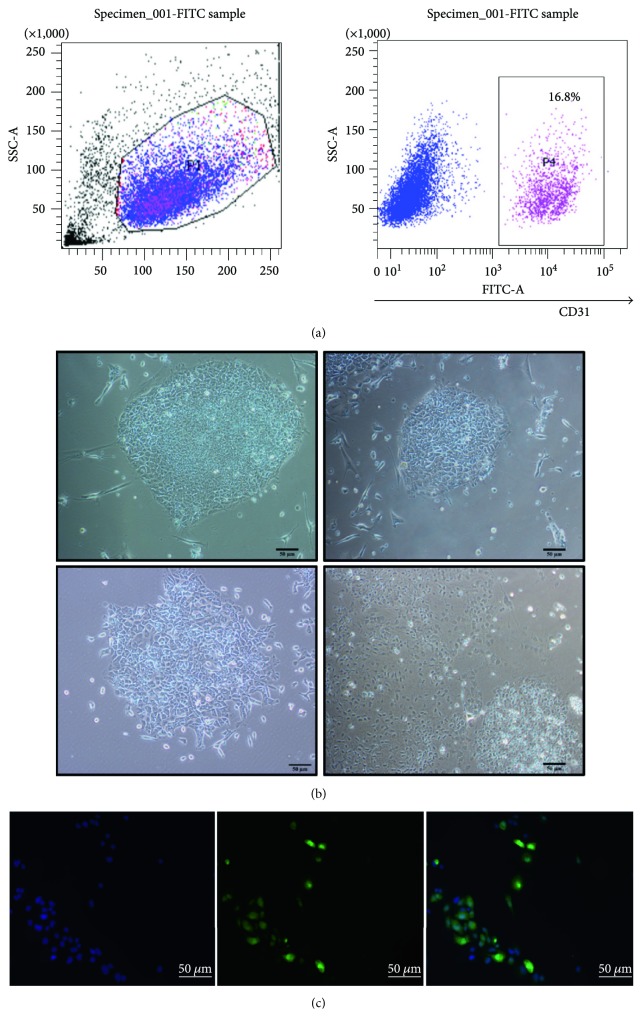
Characterization and differentiation of endothelial cells (ECs) derived from human induced pluripotent stem cells (HiPSCs). (a) FACS analysis showing the CD31 positive cells population upon differentiation protocol. FACS sorting by the endothelial markers CD31 at day 10. Efficiency to generate CD31-positive cells was 16.8%. (b) Differentiation of HiPS toward ECs. A phase-contrast image of cell appearance at 0 day (top left panel), 3 days (top right panel), 7 days (bottom left panel), and 10 days (bottom right panel) after differentiation. Scale bar = 50 *μ*m. (c) Immunofluorescent images of CD 31-positive HiPSC-ECs on day 10 during EC differentiation. DAPI was used and stained the cell nucleus. Scale bar = 50 *μ*m.

Recent findings have indicated that HiPSC-derived cells represent an ideal tool for drug testing and might hold remarkable potential in personalized regenerative cell therapies [10]. We tried to evaluate the function of HiPSC-ECs by uc001pwg.1 overexpression. Firstly, we successfully generated ECs from HiPSCs. The HiPSC-ECs were isolated by fluorescent-activated cell sorting after 10 days of differentiation and then expanded for further characterization (Figure 2(a)). The typical yield of ECs generated from the HiPSC line ranged between 11% and 20%. The expanded HiPSC-ECs formed a “cobblestone” monolayer, and immunofluorescence staining revealed that these cells were positive for endothelial marker CD31 (Figures 2(b) and 2(c)).

**Figure 3:**
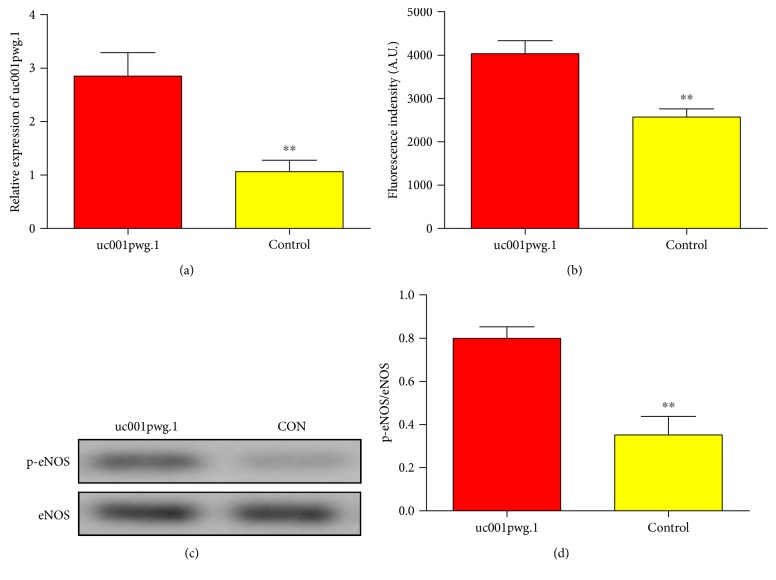
Functional assessment of HiPSC-ECs with or without viral transduction of uc001pwg.1. (a) qRT-PCR analysis of uc001pwg.1 in HiPSC-ECs as an indication of transduction efficiency. (b) The effect of uc001pwg.1 on nitric oxide (NO) generation in HiPSC-ECs using the cell-permeable fluorescent NO indicator DAF-FM. (c) Western blot analysis of endothelial nitric oxide synthase (eNOS) phosphorylation at Ser117 in HiPSC-ECs with overexpression of uc001pwg.1. (d) Bands were quantified by densitometric analysis, and the results are shown as relative density compared with control. Values expressed as mean ± standard deviation from three independent experiments. ^∗∗^*P* < 0.01.

HiPSC-ECs were then transfected with adenovirus-mediated uc001pwg.1 to upregulate uc001pwg.1 expression. The overexpression of uc001pwg.1 in HiPSC-ECs was confirmed by qRT-PCR (Figure 3(a)). Following adenovirus transduction with uc001pwg.1, these cells showed significantly higher NO production compared to controls (Figure 3(b)). The effect of uc001pwg.1 on eNOS phosphorylation was also assessed by Western blotting. We found that uc001pwg.1 significantly increased eNOS-Ser^1177^ phosphorylation in HiPSC-ECs (Figures 3(c) and 3(d)). These results suggest that uc001pwg.1 is able to functionally improve ECs derived from HiPSCs.

### 3.3. uc001pwg.1 Negatively Regulates the Expression of MCAM

**Figure 4:**
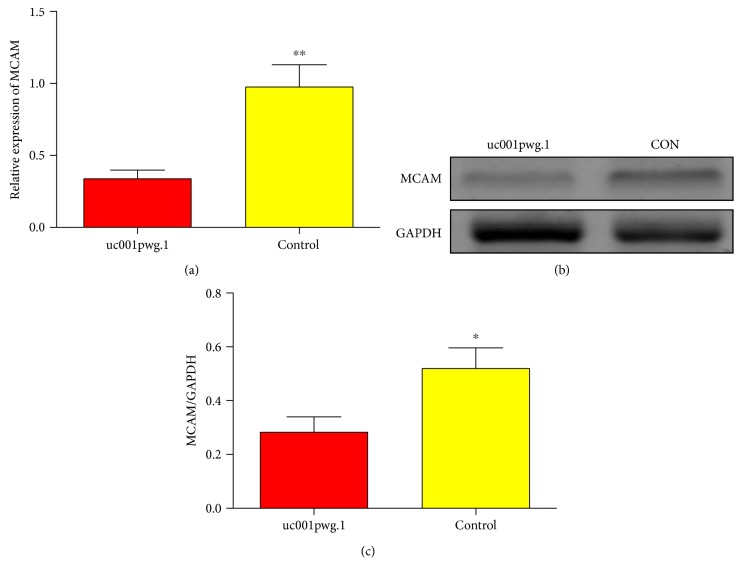
The regulatory effect of uc001pwg.1 on MCAM expression (a) The MCAM mRNA level in HiPSC-ECs after uc001pwg.1 upregulation by qRT-PCR analysis. (b) The MCAM protein level in HiPSC-ECs after uc001pwg.1 upregulation by Western blot analysis. (c) Bands were quantified by densitometric analysis, and the results are shown as relative density compared with control. Values expressed as mean ± standard deviation from three independent experiments. ^∗^*P* < 0.05 and ^∗∗^*P* < 0.01.

After confirming the effect of uc001pwg.1 on improving endothelial function, we further explored the underlying mechanism in this process. LncRNA can serve as one of the most vital intermediate phenotype on regulating mRNA expression. However, as a relatively novel kind of transcripts, the regulation relation between lncRNA and its putative target is barely known [13]. Thus, it attracted our attention to elucidate the effect of uc001pwg.1 on its associated gene RNA. On the basis of bioinformatics data, we found that MCAM is an mRNA neighboring uc001pwg.1 (exon sense-overlapping) in the lncRNA-mRNA network and is extensively implicated in a variety of oncogenic signaling transduction pathways [14]. After uc001pwg.1 upregulation, an obvious decrease in MCAM expression was observed at both mRNA and protein levels (Figure 4). Therefore, we postulate that uc001pwg.1 enhances the function of HiPSC-ECs by downregulating MCAM expression.

## 4. Discussion

Recently, a number of lncRNAs have been identified as an important controller of cellular functions via regulating RNA transcription, degradation, and translation. In the present study, the expression profiles of lncRNAs in human stenosed and nonstenotic uremic veins were examined via RNA-sequencing analysis. Long noncoding uc001pwg.1, originally discovered by UCSC_knownGene, is located on chromosome 11 with a length of 441 bps. Our current study found that lncRNA uc001pwg.1 was found enriched in both control vein segments and HUVECs and was emphasized via qRT-PCR validation as a consequence. Furthermore, HiPSC-ECs that have broader prospects in medical application were used in our research. We revealed that upregulated uc001pwg.1 enhanced the function of HiPSC-ECs. Meanwhile, our results presented that uc001pwg.1 overexpression led to the downregulation of MCAM expression, which showed a novel mechanism by which uc001pwg.1 played a vital role in endothelial function through mediating the expression of MCAM.

Our data clearly showed that effects of lncRNA uc001pwg.1 on the function of HiPSC-ECs by overexpressing the lncRNA. DAF-FM diacetate assay results indicated that NO production was promoted in HiPSC-ECs upon uc001pwg.1 overexpression. Additionally, we found that enhanced uc001pwg.1 could increase the eNOS phosphorylation in HiPSC-ECs. Mounting evidence suggests that the hallmark of endothelial dysfunction is the reduction in the bioavailability of NO [15, 16]. Early processes involved with both expansive and constrictive vascular remodeling are usually mediated by vasomotor changes. In parallel with the importance of NO for initiating vasodilatory responses in the coronary and skeletal muscle circulations, mechanisms affecting its bioavailability are critical during vascular remodeling as crucial determinants of the final lumen size [17]. eNOS, the last of the three mammalian NOS isoforms to be isolated, was originally purified and cloned from vascular endothelium [18]. The ability of eNOS to generate NO allows for control of vascular tone along with preventing inflammation and proliferation of VSMCs in the subendothelium [19]. Alterations in eNOS activity or expression are linked to a number of cardiovascular pathologies that exhibit endothelial dysfunction. Phosphorylation/dephosphorylation of eNOS appears to be a major factor in the regulation of eNOS activity [20]. Therefore, eNOS phosphorylation and NO production of cells were detected to evaluate EC function in the present study. Our findings provide evidence that uc001pwg.1 functions as a key mediator of endothelial function. Like atherosclerosis, the neointima that develops in AVF has preferentially occurred in areas of low fluid shear stress and oscillatory flow [21]. It should be noted that hemodynamic stress plays a significant role in determining the functional phenotype of the vascular endothelium. We could extend our targeted cell delivery strategy to the use of HiPS-ECs that overexpress uc001pwg.1 for targeted cell therapy in animal AVF model in the future.

In view of the data gathered from these databases, the following investigation focused on the endothelial transmembrane protein MCAM, which is the nearby gene of the uc001pwg.1. MCAM and has been thoroughly studied and found to be physiologically expressed on different cells in the organism, as the subset of T lymphocytes Th17 and vascular cells including ECs [22, 23]. In particular, it has been identified that MCAM is a major component of the endothelial junction, controlling cell-cell cohesion, paracellular permeability, inflammatory response, and angiogenesis [24, 25]. A current study supports a role for MCAM as an essential gene for renal EC development [26]. It has been established that endothelial dysfunction (ED) occurs after coronary artery bypass grafting (CABG). A recent report shows increased concentrations of MCAM 3 months after CABG [27]. Some researchers have confirmed a role of MCAM as an endothelial cell dysfunction marker in diabetic patients [28, 29]. Thus, MCAM is considered a candidate gene involved in ED. In our study, we tried to make a clarification of lincRNA-related mechanisms underlying the functional improvement of HiPS-ECs. Interestingly, we confirmed that ectopic uc001pwg.1 expression caused a dramatic decrease of MCAM expression. These results indicate that uc001pwg.1 modulates endothelial function, at least in part, by regulating MCAM expression.

## 5. Conclusions

In summary, our study is the first to show that lncRNA uc001pwg.1 plays an important role in endothelial dysfunction in stenotic lesions of AVF failure. After successful generation of ECs from HiPSCs, we found that enforced uc001pwg.1 could increase eNOS phosphorylation and NO production in vitro. And the improvement of endothelial function may be related to MCAM downregulation. The strategy utilizing HiPS-ECs overexpressed uc001pwg.1 appears to be a potential for translation to the treatment of neointima formation and failed AVF in future animal studies.

## Supplementary Materials

Supplementary 1 Clinical characteristics of two groups.

Supplementary 2 A versus N up- or downregulated transcripts.

Supplementary 3 Go analyses of the differentially expressed IncRNAs.
