# Supplementary material for: Long Noncoding RNA uc001pwg.1 Is Downregulated in Neointima in Arteriovenous Fistulas and Mediates the Function of Endothelial Cells Derived from Pluripotent Stem Cells
Source: Stem Cells Int. 2017 Dec 13;2017:4252974. doi: 10.1155/2017/4252974 (PMC5745761; doi:10.1155/2017/4252974)
Supplement: Supplementary 1 — Clinical characteristics of two groups. [file 4252974.f1.doc]

**Table 1. Clinical characteristics of two groups**

|  | **Stenosis** | | **Control** | **P value** |
| --- | --- | --- | --- | --- |
|  | **N=4** | | **N=3** |  |
| Age (years) | | 53±5.60 | 52±7.94 | 0.426 |
| Males/females | | 2/2 | 1/2 | 0.659 |
| Serum creatinine (μmol/L) | | 746±96.51 | 711.33±68.41 | 0.311 |
| Blood urea nitrogen (mmol / L) | | 23±6.98 | 22±6.56 | 0.428 |
| Duration of hemodialysis (years) | | 2.5±1.29 | 2±1.00 | 0.302 |
| Hypertention | | 1 | 1 | 0.809 |
| Diabetes mellitus | | 1 | 1 | 0.809 |
| Cerebral disease | | 0 | 0 | / |
| Peripheral ischemic disease | | 0 | 0 | / |
| Secondary hyperparathyroidism | | 0 | 0 | / |
| Chronic glomerulonephritis | | 0 | 0 | / |
